# Redescription of the dasydytid gastrotrich *Haltidytesooëides* (Brunson, 1950) based on type material

**DOI:** 10.3897/zookeys.785.28382

**Published:** 2018-09-19

**Authors:** Axell K. Minowa, André R. S. Garraffoni

**Affiliations:** 1 Laboratório de Evolução de Organismos Meiofaunais, Departamento de Biologia Animal, Instituto de Biologia, Universidade Estadual de Campinas, 13083-970, Campinas, São Paulo, Brazil Universidade Estadual de Campinas Campinas Brazil

**Keywords:** Chaetonotida, Dasydytidae, Gastrotricha, *
Haltidytes
*, USA

## Abstract

The semi-pelagic gastrotrich species *Haltidytesooëides* (Brunson, 1950) is redescribed based on original type material deposited at the Smithsonian National Museum of Natural History. Herein we present a new diagnosis and figures of the species, detailing the insertion position of the lateral spines, misinterpreted in the original description. Furthermore, we reassess the taxonomic key for the genus *Haltidytes* Remane, 1936 based on our new findings.

## Introduction

While most gastrotrichs are epibenthic, periphytic, or interstitial, some species belonging to the family Dasydytidae Daday, 1905 present a semi-pelagic lifestyle (Kieneke et al. 2008, Balsamo et al. 2014, Kånneby and Todaro 2015). Seven genera are currently assigned to Dasydytidae, including the genus *Haltidytes* Remane, 1936 recently found as monophyletic (Minowa and Garraffoni 2017). *Haltidytes* was originally established as a subgenus of *Dasydytes* Gosse, 1851 by Remane (1936), who then elevated it to a genus rank (Remane, 1967). Currently, the genus *Haltidytes* contains six valid species (Minowa and Garraffoni 2017): *H.festinans* (Voigt, 1909) (type species), *H.crassus* (Greuter, 1917), *H.ooëides* (Brunson, 1950), *H.saltitans* (Stokes, 1887), *H.squamosus* Kisielewski, 1991, and *H.pseudosquamosus* Minowa & Garraffoni, 2017.

While preparing a forthcoming study incorporating phylogenetic analyses of all valid Dasydytidae species based on morphology (Minowa and Garraffoni, in preparation), we came across the possible type specimen of *Haltidytesooëides* (Brunson, 1950), originally described as *Dasydytesooëides* (USNM W 26869S). Although Brunson (1950) had not designated any type specimen, the locality and sampling date (Michigan State, Washtenaw County, Half-Moon Lake; May, 30, 1944) registered in the Smithsonian Data Base are the same as those reported in the Brunson’s study. It came as a surprise to us that after more than 75 years the specimen is still preserved. Due to small size and fragile bodies, fixed specimens of gastrotrichs usually have their diagnostic morphological characters deteriorated after fixation (e.g. Balsamo et al. 2014, Kånneby 2016, Garraffoni and Freitas 2017). It is interesting to highlight that we also found the possible type specimen of *Stylochaetascirtetica* Brunson, 1950 (USNM W 26870), but in this case, the material is in a poor condition and could not be used for a reanalysis.

## Materials and methods

Herein we redescribe *Haltidytesooëides* based upon a single type material deposited at the Smithsonian National Museum of Natural History. External morphology was observed using an Olympus BX63F compound fluorescence microscope with a digital DP80 camera and cellSens software (Olympus, Philadelphia, USA). Videos were prepared using the open-source platform Fiji (Schindelin et al. 2012). The necessity for a re-examination was caused by the shallow description by Brunson (1950), who only briefly reported and illustrated a few morphological features of the new species. This need was further noted by Balsamo et al. (2014), suggesting a misinterpretation of the insertion positions of the lateral spines. In the redescription of the species, the groups of spines are coded according to Kisielewski (1991).

## Taxonomic account

### Phylum Gastrotricha Metschnikoff, 1865

#### Order Chaetonotida Remane, 1925 [Rao & Clausen, 1970]

##### Family Dasydytidae Daday, 1905

###### Genus *Haltidytes* Remane, 1936

####### Haltidytes ooëides

Taxon classificationAnimaliaChaetonotidaDasydytidae

 (Brunson, 1950)

Figs 1 , 2

######## Redescription.

**Figure 1.**
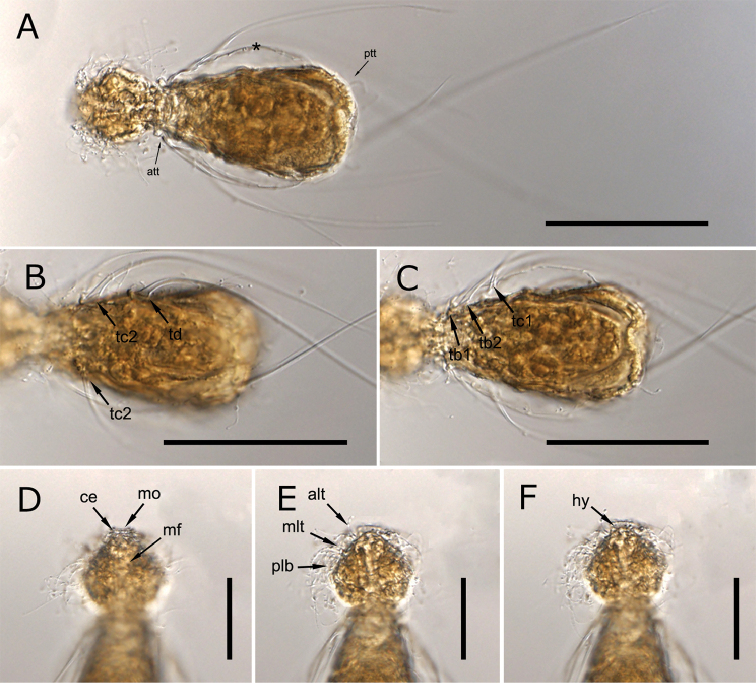
Light micrographs (DIC) of *Haltidytesooëides* (Brunson, 1950). **A** dorsal view, **B–C** ventral view showing the insertion of trunk spines **D** dorsal view of the head **E–F** ventral view of the head. Asterisks (*) indicate the body cuticle. Scales bars: **A–C** 50 µm, **D–F** 25 µm. Abbreviations: **alt** anterior lateral ciliary tuft **att** anterior locomotory ciliation tuft **ce** cephalion, **hy**: hypostomion **mf** middle furrow **mlt** mediolateral ciliary tuft **mo** mouth **plb** posterior lateral ciliary band **ptt** posterior locomotory ciliation tuft **tb1-2** trunk spines **tc1-2** trunk spines **td** trunk spines.

**Figure 2.**
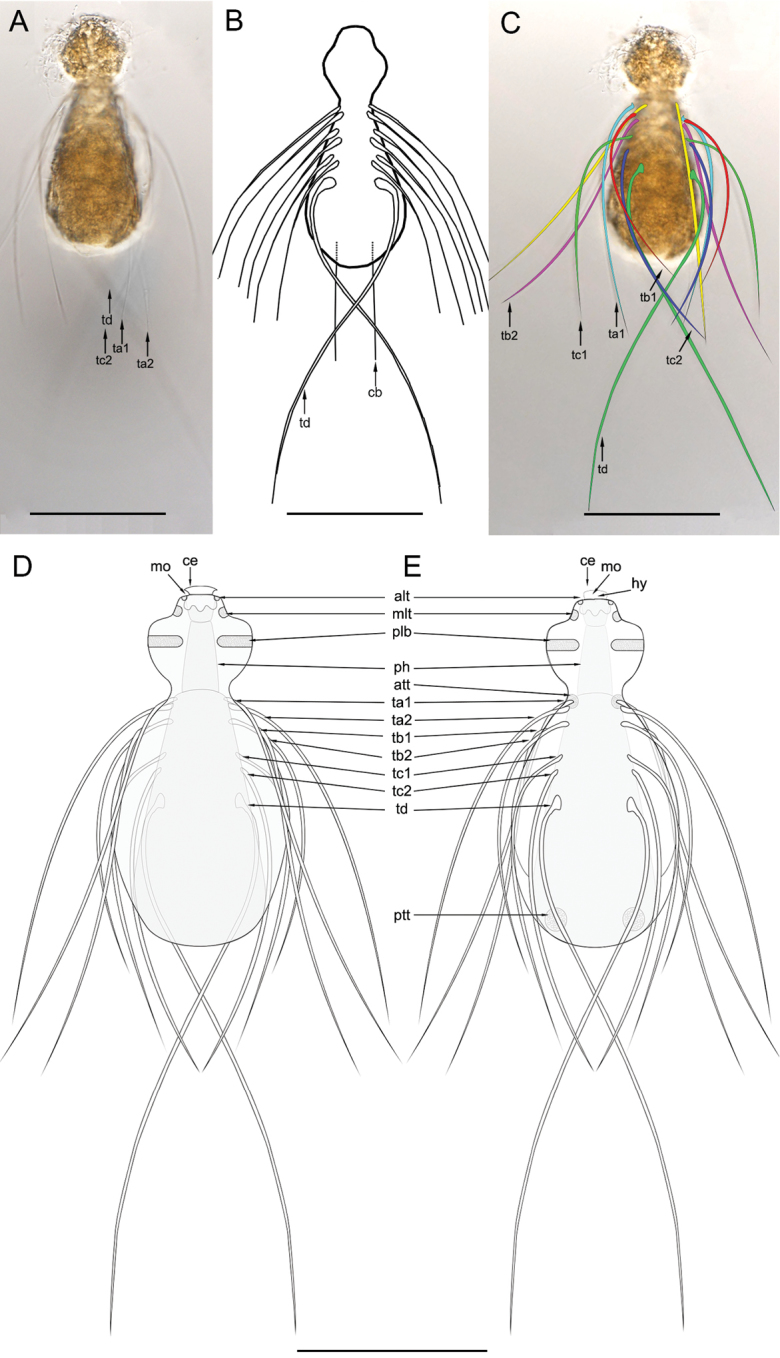
Light micrographs (DIC) and redrawing of the schematic drawing of *Haltidytesooëides* (Brunson, 1950). **A** whole animal view **B** redrawing of the schematic drawing of the original description **C** the same image of **A** in which the arrangement of the trunk spines is highlighted **D** schematic drawing of the dorsal view **E** schematic drawing of the ventral view. Scales bars: 50 µm. Abbreviations: **alt** anterior lateral ciliary tuft **att** anterior locomotory ciliation tuft **cd** caudal bristle **ce** cephalion **hy**: hypostomion **mlt** mediolateral ciliary tuft **mo** mouth **ph** pharynx **plb** posterior lateral ciliary band **ptt**: posterior locomotory ciliation tuft **ta1-2** trunk spines **tb1-2** trunk spines **tc1-2** trunk spines **td** trunk spines.

The observed specimen has a compact, bowling pin-shaped body, measuring 88 μm in total body length, 184 μm with spines included. Conical head with convex sides (24 μm wide), pentalobate, dorsally with a middle furrow (Figure 1D). Cephalic ciliature consists of two lateral tufts, one adjacent to mouth and another slightly posterior, and a transverse band interrupted medially in the ventral and dorsal portion on middle head region (Figures 1E, 2C, D). Kephalion, trapezoidal in shape (9 μm length, 13 μm wide) (Figures 1D, 2C), hypostomion, triangular in shape (5 μm length, 8 μm wide) located around the ventral portion of the mouth ring (Figures 1F, 2D). Distinct neck constriction (17 μm wide), much narrower than the head and trunk. Trunk ovoidal in shape (42 μm in maximum width) with a rounded posterior end (Figure 1A).

Cephalic spines or rear spines not observed. On the anterior half of the trunk four paired groups of 2-2-2-1 curved simple spines (ta1-2, tb1-2, tc1-2, td) respectively, inserted directly on the cuticle without scales (Figs 1B, C, 2A–D). The first group (ta1 at U32; ta2 at U35) inserted ventrolaterally at the neck base strongly bends dorsally at the neck level showing a slightly (almost straight) concave curvature extending all over the trunk (Figure 2B). The other three groups (tb, tc and td) are inserted ventrally at U32, U35, U38, U40, U46, U50, and U60, respectively (Figure 1 B–C, 2 B). Spines tb2 turn dorsally like spines ta. Spines tb1, tc1-2, and td show a slight convex curvature and extend ventrally along the trunk, (Figure 2B). Spines of ta to tc group measure 100, 90, 75, 90, 82, 80 μm respectively. Group td is composed of one pair of very long saltatorial spines, 140 μm in length.

Trunk locomotory ciliation divided into 2-paired ventral tufts at 15U and 93U on the ventral side of at the neck and posterior trunk, respectively (Figure 2C, D). No dorsal sensory bristles were observed.

Mouth ring is terminal (3 μm in diameter). Pharynx (33 μm in length) increases in width uniformly from 9 μm anteriorly to 11 μm at the posterior end) (Figure 1A, D, F).

######## Remarks.

Usually, the trunk width is given as the maximum trunk widthwhich is at the midgut level. In this case, the type specimen *H.ooëides* is 42 μm wide. However, Brunson (1950) measured the trunk width posterior to the midgut level (close to the posterior end of the body) and found it to be 36 μm wide.

Differences in spines length between the original description and the present one (Table 1) are due to different measurement methods. We chose to measure each spine length outlining its curvature (100, 90, 75, 90, 82, 80 μm respectively) instead of measuring the distance between the spine base insertion and apex as a straight line, as Brunson (1950) did (86, 86, 67, 82, 82 and 58 μm, respectively).

Additionally, the original description mentions a pair of caudal bristles (Figure 2C) that originate 10 µm from the posterior end of the trunk. After reexamination of the type specimen (Figure 2A, B–D) we conclude that Brunson (1950) may have misinterpreted these structures. In fact, our observations revealed that the caudal bristles described by Brunson (1950) actually are the ta1 spines, due to their similar position relative to the posterior trunk, size and shape (Figure 2A, B).

As previously mentioned, the description of some morphological characters of *H.ooëides* were misinterpreted by Brunson (1950) and incorrectly replicated by Balsamo et al. (2014) and Minowa and Garraffoni (2017). We address this issue by correcting the taxonomic key *Haltidytes* in order to correct previous misinterpretations.

**Table 1.** Morphometric features of *Haltidytesooëides* (Brunson, 1950): measures are expressed in µm; the relative positions of morphological structures along the body are expressed in percentage unities (U) in relation to the total body length.

Feature type	Measure (μm)
Total body length, spines excluded	88
Total body length, spines included	184
Maximum head width) (U17)	24
Minimum neck width (µm) (U30)	17
Maximum trunk width (µm) (U65)	42
Trunk length (µm)	60
Pharynx length (µm)	33
Anterior pharynx width (µm)	9
Posterior pharynx width (µm)	11
Diameter of mouth ring (µm)	3
Kephalion length (µm)	9
Kephalion width (µm)	13
Hypostomion length (µm)	5
Hypostomion width (µm)	8
Spine ta1-2 length (µm)	100, 90
Spine tb1-2 length (µm)	75, 90
Spine tc1-2 length (µm)	82, 80
Spine td length (µm)	140
Ventral spine ta1-2 insertion	U32, U35
Ventral spine tb1-2 insertion	U38, U40
Ventral spine tc1-2 insertion	U46, U50
Ventral spine td insertion (U)	U60
Cephalic ciliary tufts insertion (U)	U1, U4, U8
Ventral trunk ciliary tufts insertion (U)	U15, U93

### Taxonomic key to genus *Haltydytes*

**Table:** 

1	Seven pairs of spines with ventral insertion besides td (saltatorial spines)	**2**
–	Six or fewer pairs of spines with ventral insertion besides td (saltatorial spines)	**4**
2	Dorsal trunk totally or partially covered with rhombic scales arranged sparsely or aggregate	**3**
–	Dorsal trunk without scales	*** H. festinans ***
3	Dorsal scales arranged sparsely; dorsal trunk covered with rhombic scales with a short median keel or smooth; ventral trunk covered with small smooth scales	*** H. squamosus ***
–	Dorsal scales aggregate; median and rear dorsal trunk covered with smooth, rhombic scales; ventral trunk without scales	*** H. pseudosquamosus ***
4	Anterior spines (ta) cross each other above dorsal trunk	*** H. saltitans ***
–	Anterior spines (ta) do not cross each other above dorsal portion of the trunk	**5**
5	Ventral ciliature consisting of 2 tufts; 3 pairs of spines arrive to the dorsum while 3 other spines remain ventral besides td group	*** H. ooëides ***
–	Ventral ciliature consisting of 2 longitudinal bands; all 5 pairs of spines arrive to the dorsum; only td group remain on venter	*** H. crassus ***

## Supplementary Material

XML Treatment for Haltidytes ooëides
